# SNP-PRAGE: SNP-based parametric robust analysis of gene set enrichment

**DOI:** 10.1186/1752-0509-5-S2-S11

**Published:** 2011-12-14

**Authors:** Jaehoon Lee, Soyeon Ahn, Sohee Oh, Bruce Weir, Taesung Park

**Affiliations:** 1 Department of Statistics, Seoul National University, San 56-1, Shilim-dong, Seoul, Korea; 2 Medical Research Collaborating Center, Seoul National University Bundang Hospital, 166 Gumi-ro, Bundang-gu, Seongnam 463-707, Korea; 3 Department of Biostatistics, University of Washington, Box 357232, Seattle, Washington 98195, USA

## Abstract

**Background:**

The current genome-wide association (GWA) analysis mainly focuses on the single genetic variant, which may not reveal some the genetic variants that have small individual effects but large joint effects. Considering the multiple SNPs jointly in Genome-wide association (GWA) analysis can increase power. When multiple SNPs are jointly considered, the corresponding SNP-level association measures are likely to be correlated due to the linkage disequilibrium (LD) among SNPs.

**Methods:**

We propose SNP-based parametric robust analysis of gene-set enrichment (SNP-PRAGE) method which handles correlation adequately among association measures of SNPs, and minimizes computing effort by the parametric assumption. SNP-PRAGE first obtains gene-level association measures from SNP-level association measures by incorporating the size of corresponding (or nearby) genes and the LD structure among SNPs. Afterward, SNP-PRAGE acquires the gene-set level summary of genes that undergo the same biological knowledge. This two-step summarization makes the within-set association measures to be independent from each other, and therefore the central limit theorem can be adequately applied for the parametric model.

**Results & conclusions:**

We applied SNP-PRAGE to two GWA data sets: hypertension data of 8,842 samples from the Korean population and bipolar disorder data of 4,806 samples from the Wellcome Trust Case Control Consortium (WTCCC). We found two enriched gene sets for hypertension and three enriched gene sets for bipolar disorder. By a simulation study, we compared our method to other gene set methods, and we found SNP-PRAGE reduced many false positives notably while requiring much less computational efforts than other permutation-based gene set approaches.

## Background

The genome-wide association (GWA) studies have been successful to investigate generic variants associated with some targeted phenotypes. In general, many GWA methods only consider association of a single SNP and provide the list of the most significant SNPs or related genes due to computational burden.

However, complex diseases often result from compound action of multiple risk factors and therefore the single-SNP-based analysis may miss the genetic variants that affect risk effects jointly but have scarce individual effects. Also, the locus heterogeneity, which implies that alleles at different loci target the same diseases in different individuals, would increase difficulty in replication of association of a single marker [1]. Furthermore, a large number of statistical tests may result in high false positive associations [2]. To resolve these issues, it was suggested to utilize prior biological knowledge or known pathway information, and thus to incorporate a set of related SNPs, which leads a smaller number of tests. This approach was motivated by the gene set analysis (GSA), widely used in the analysis of microarray data. GSA focuses on gene sets rather than individual genes, and combines weak signals from a number of individual genes in a set, when individual genes are weakly associated with the traits. In this way, GSA increases a power of detecting disease-related genes and helps to interpret underlying genetic background and has been popularized.

GSA can be classified into non-parametric or parametric approach. The most popular non-parametric GSA method is gene set enrichment analysis (GSEA) [3]. GSEA uses the enrichment score which represents whether the members of gene set tend to occur toward top or bottom in ranked gene list based on a correlation. It permutes the phenotype label and repeats calculating the enrichment score for the test. This requires very expensive computational efforts.

On the other hands, the parametric GSA can reduce computing time by assuming a specific distribution. A hypergeometric distribution-based test [4,5] is a typical choice for the parametric method, and binomial, normal, and chi-square distributions are also widely used [6-8].

There are several prior works for applying GSA methods to GWA data [1,2,7,9-15]. For simplicity, we call all these methods as GSA-GWA. We address two issues regarding GSA-GWA. The first issue is that there has not been a widely agreed and accepted theory on how to combine the measures of multiple SNPs into one single gene-level measure, and moreover how to combine the gene-level measures into one single gene-set level measure. In original GSA, the gene-level measure is typically a fold-change or a correlation to represent the effect of a single gene. In GWA data, however, it is often required to calculate association measures of genes by combining the SNP-level measures. The SNP-level measures include p-values, or chi-square test statistics from the univariate SNP-to-phenotype association tests. Once the SNP-level measure is decided, the gene-level summary statistics are then derived as the highest SNP-level statistics [10], the sum of SNP-level statistics [9], or the combined p-value [1].

However, there are some substantial limitations in current GSA-GWA methods. First, in deriving the summary statistics the correlation among the SNP-level association measures has not been taken appropriately into account which is expected to play an important role. The SNP-level association measures are usually correlated because the linkage equilibrium (LD) exists among SNPs. If this correlation is not correctly adjusted, the resulting gene-set-level measure would be inflated [1]. Unfortunately, many GSA-GWA methods have not considered the LD structures adequately.

Second, the computational burden is heavy. Once having the gene-level association measures, it is possible to apply different GSA methods to get various gene-set-level statistics and evaluate their performances. However, as explained later, the majority of GSA-GWA methods implement non-parametric permutation to calculate the observed significance, which takes a heavy computing time.

There have been several efforts to resolve these limitations. As the pioneering work of GSA-GWA, GSEA [3] was extended to GWA data by Wang *et al*. [10], which has been implemented in GenGen package [11] It repeats permutation of sample label and calculation of gene set statistics 100~1,000 times [2,10,12-14]. This permutation-based testing can preserve a correlation among the SNP-level measures, but this is very computationally expensive in genome-wide scale.

In order to reduce computing time, some GSA-GWA studies use a parametric test. Peng *et al.*[1] used various kinds of the parametric test such as Fisher’s combination test, Sidak’s combination test, Simes’ combination test, and a FDR-based test under the independence assumption of the SNP-level p-values. A GLOSSI method developed by Chai *et al.*[9] used Fisher’s combination test under the assumption of correlated p-values.

Recently, Nam *et al*. [15] proposed the Z-statistic method that compares a specific gene-set to others. This method is the extension of the parametric analysis of gene set enrichment (PAGE) [7], which is the parametric and competitive GSA for microarray data. PAGE uses the mean of the association measures in a set as a summary measure and assumes that it follows a normal distribution by the central limit theorem when the number of genes is large.

However, these parametric methods including the Z-statistic method do not consider the LD structures adequately and assume no correlation between SNP-level p-values. In order to overcome these limitations of current GSA-GWA, we propose SNP-PRAGE, a SNP-based parametric robust analysis of gene-set enrichment, which is based on a simple normality assumption. SNP-PRAGE estimates the LD information among SNPs based on haploblock-wise covariance structure to consider the correlation among SNP-level measures without taking the permutation step.

We compare our method to other GSA-GWA methods via the simulation study in terms of size, power and computing time. We also demonstrate SNP-PRAGE using two GWA data sets: hypertension data of 8,842 samples from the Korean population and bipolar disorder data of 4,806 samples from the Wellcome Trust Case Control Consortium (WTCCC).

## Methods

### Z-statistic method (GSA-SNP)

Nam *et al*. [15] implemented the Z statistic method in their software, GSA-SNP. The negative logarithm of the *m*th best p-value within each gene was used as the gene summary measure. Based on this gene summary measure, the Z-score was then calculated as gene-set-level summary. The Z-score was assumed to follow a normal distribution based on the central limit theorem (CLT).

**Figure 1:**
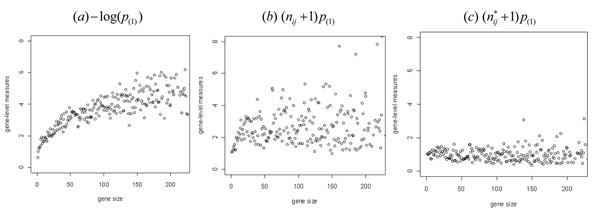
Distribution of gene-level measures over the gene size for hypertension data from Korean population. The x-axis is gene size which is a number of SNPs within the gene and the y-axis is mean of gene-level summaries with same gene size.

In order to meet a normal distribution assumption, the gene-level order statistic is assumed to have an identical and independent distribution (i.i.d.). Let *n_ij_* be the gene size which is the number of SNPs within the *j*th gene in the *i*th gene set. If we assume a p-value follows an independent uniform distribution, the *m*th order p-value *p*_(_*_m_*_)_ follows a beta distribution with the mean *m*/(*n_ij_*+1) and the variance *m*(*n_ij_*–*m*+1) /{(*n_ij_*+1)^2^(*n_ij_*+2)}. This means that the gene with many SNPs have a lower *p*_(_*_m_*_)_ than genes with a few SNPs. (See Figure 1(a)). So *p*_(_*_m_*_)_ is not identically distributed over the gene size. To satisfy the identical distribution assumption, the summary measures need some modifications.

The gene-level summary measure is also assumed to have a homogeneous variance. However, the variance of their summary measures also depends on the gene size. When the gene size is large, the variance of the summary measure of the gene tends to be small. This problem can be easily addressed by modifying Welch’s *t* statistic [20] which is designed to handle for the heterogeneous variance of the two groups.

### SNP-PRAGE

To address these issues of the Z-statistic method we mentioned above, we multiply *p*_(_*_m_*_)_ by (*n_ij_*+1) to have an approximate identical distribution over the gene size. The moment generating function of (*n_ij_*+1)*p*_(_*_m_*_)_ does not depend on the gene size *n_ij_* when p-values are independent from each other and *n_ij_* is large enough. However, the SNP-level p-values are not independent from each other because of the LD structure. So (*n_ij_*+1)*p*_(_*_m_*_)_ has a non-identical distribution over the genes (See Figure 1(b)).

In SNP-PRAGE, we propose using the effective gene size  instead of gene size *n_ij_* to make sure that the gene-level summary measure has an approximate identical distribution over the gene size irrespectively of correlation among p-values. The effective gene size is computed by using the following equation.

 is estimated under the independent covariance structure and  under the haploblock-wise compound symmetric covariance structure.

Note that SNP-level measures within a LD block are correlated. The within-gene covariance matrix can be estimated by using maximum likelihood (ML) estimation. Among the several candidate covariance structures, the Akaike information criterion is used to choose the most appropriate covariance structure [16]. First, we construct the LD block among SNPs in GWA data so that any pair of SNPs from different LD blocks is independent from each other (r^2^≤ 0.05) [17]. Second, we obtain the ML estimator of the covariance matrix within the LD block for each gene set. The most appropriate covariance structure is then selected via AIC. In the Korean GWA data analysis, the LD-block-wise compound symmetric structure (LD-CS) was chosen as the appropriate covariance structure.

Within the gene, the highly ranked p-values tend to be correlated because of the LD structure. Through the simulation study, we found that the average of the top *m* p-values from a gene is a more robust gene-level summary measure than only the *m*th p-value (data are not shown). The following is the final gene-level summary measure proposed in SNP-PRAGE. In Figure 1(c), we can see this measure has the identical mean over the gene size.

**Figure 2:**
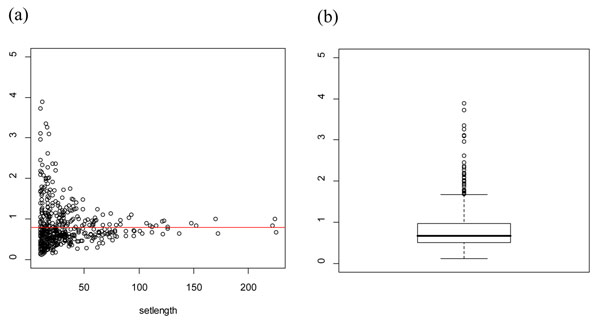
Variance of gene-level measure over the gene sets. In the left plot (a), x-axis is gene set size (= number of genes in the gene set) and y-axis is sample variance of gene-level summaries in the gene set. The right plot (b) shows a boxplot of variance of gene-level measures. A red line represents total sample variance in the data.

However, our empirical study shows that gene-level measure  does not have the common variance over the gene set especially with the small gene set size (See Figure 2). Thus, we assume that the gene-level measure has a heterogeneous variance over the gene sets:

The mean of the gene-level measures in a gene set follows a normal distribution by the central limit theorem.

We compute the sample variance distinctly over gene set and derive the following set-level test statistic:

The degree of freedom (*df*_i_) is computed by Welch-Satterthwaite equation [20].

## Results

### Hypertension data from the Korean GWA study

We used canonical pathways from MsigDB database [18]. These canonical pathways are curated from other online database such as BioCarta, KEGG and GO and so on. MsigDB database contains 639 pathways and 4934 genes.

We applied SNP-PRAGE to GWA data set from the Korean GWA study which was initiated in 2007 to undertake a large-scale GWA analysis among 10,038 participants (aged between 40 and 69) of Ansung (n=5,018) and Ansan (n=5,020) population-based cohorts [19]. These cohorts, established as part of the Korean Genome Epidemiology Study (KoGES) in 2001 provide extensive phenotypic data for over 260 traits, but here we focus on analyses of hypertension. From the total of 10,038 participants, DNA was available for 10,004, all of whom were genotyped with the Affymetrix Genome-Wide Human SNP array 5.0 and the Bayesian Robust Linear Modeling using Mahalanobis Distance (BRLMM) algorithm. Markers with high missing gene call rate (>5%), low MAF (<0.01) and significant deviation from Hardy-Weinberg equilibrium (P < 1 × 10^-6^) were excluded, leaving 352,228 SNPs. After removing samples with low call rates (< 96%, *n* = 401), sample contamination (*n* = 11), gender inconsistencies (*n* = 41), cryptic relatedness (*n* = 608) and serious concomitant illness (*n* = 101), GWA genotypes from 8,842 individuals were included. Hypertension phenotype was defined as a systolic blood pressure (SBP) ≥ 140 mm Hg or a diastolic blood pressure (DBP) ≥ 90 mm Hg. The logistic regression analysis with an additive model (1 *d.f.*) is conducted after adjustment for age, sex, and recruitment area (i.e. Ansung and Ansan). To correct for stratification, some methods that infer genetic ancestry, such as principal component analysis (PCA) and structured association can be used [21]. In our GWA data, there is no evidence of population stratification.

We obtained the SNP ID, rs ID, position information from dbSNP build 128 and gene ID, gene name, and position information from NCBI build 36. Each SNP is mapped to a gene closest to it. Only SNPs located within 500 Kb upstream or downstream of a gene are considered, because most enhancers and repressors are less than 500Kb away from genes, and most LD blocks are within 500Kb [10]. As a result of mapping it covered 60% of all SNPs in our data. If the mapping range is larger, we could save more SNPs, but the risk of SNP’s mapping to shared region of overlapping genes also increases.

Our proposed SNP-PRAGE was used to identify the significant gene sets associated with hypertension in Korean GWA data. We used the p-value from the logistic regression as SNP-level association measure for each SNP. We compared three kinds of gene-level measures, , , and 

In Figure 1, each plot shows the mean of gene-level measures over the gene size. Figure 1(a) is from the gene-level measure used in the Z-statistic method. This measure tends to increase as the gene size increases. The non-causal gene set which has a larger number of genes tend to be detected as significant. Figure 1(b) shows the minimum p-values within the gene multiplied by (gene size + 1) over the gene size. Figure 1(c) shows the same plot but uses the effective gene size instead of the actual gene size. Figure 1(c) is most robust to the gene size showing the constant pattern.

Next, we checked the homogeneity assumption of variance of gene-level measure () over the gene sets. Figure 2 represents whether  has the homogeneous variance over the gene sets. We can see the sample variances are different over the gene sets especially for the gene sets with a small number of genes. Thus, it would be inappropriate to assume the homogeneous variance assumption for the gene-level measures. SNP-PRAGE allows the heterogeneous variance of gene-level measure.

In order to handle multiple testing problems, the false discovery rate (FDR) was controlled [22]. The q-values were calculated to guard against the cost of multiple hypothesis testing [23]. The q-value provides an expected proportion of false positives among sets with unadjusted p-values at least as extreme as the current set of interest. Single SNP association test based on a logistic regression cannot detect SNP whose q-value is less than 0.05. Minimum SNP-level p-value is 2.043 × 10^-6^ and corresponding q-value is 0.4. Even though there is no significant SNP-level association in terms of q-values, multiple SNPs with moderate effects could affect the phenotype in the gene set-level.

Table 1 and Table 2 summarize the top 5 gene sets obtained by using the Z-statistic method and SNP-PRAGE, respectively. In Z-statistic method, minimum q-value is 0.06, which is not significant if we use 0.05 as q-value cut-off. SNP-PRAGE yielded 2 significant gene sets (q-values: 0.01, 0.03) based on q-value 0.05 as cut off, while Z-statistic method did not yield any significant gene sets.

**Table 1:** KARE result: top 5 gene sets with smallest q-value associated with hypertension phenotype from Z-statistic method

Gene set	No. genes	No. SNPs	p-value	q-value
ST_JNK_MAPK_PATHWAY	36	2410	1.13 × 10^-4^	6.38 × 10^-2^
HSA00563_GLYCOSYLPHOSPHATIDYLINOSITOL_ANCHOR_BIOSYNTHESIS	18	700	2.67 × 10^-4^	6.57 × 10^-2^
FASPATHWAY	28	1489	8.82 × 10^-4^	1.44 × 10^-1^
HSA05060_PRION_DISEASE	117	762	2.42 × 10^-3^	2.53 × 10^-1^
HSA04520_ADHERENS_JUNCTION	64	4150	2.58 × 10^-3^	2.53 × 10^-1^

**Table 2:** KARE result: top 5 gene sets with smallest q-value associated with hypertension phenotype from SNP-PRAGE

Gene set	No. genes	No. SNPs	p-value	q-value
ST_JNK_MAPK_PATHWAY	36	1701	2.40 × 10^-5^	9.48× 10^-3^
ST_ERK1_ERK2_MAPK_PATHWAY	24	1765	1.61 × 10^-4^	3.16 × 10^-2^
HSA05214_GLIOMA	52	2301	3.92 × 10^-4^	5.16 × 10^-2^
HSA05050_DENTATORUBROPALLIDOLUYSIAN_ATROPHY	14	997	7.97 × 10^-4^	7.57 × 10^-2^
EXTRINSICPATHWAY	13	579	9.58 × 10^-4^	7.57 × 10^-2^

The significant gene sets in SNP-PRAGE are ST_JNK_MAPK_Pathway and ST_ ERK1_ERK2_MAPK_Pathway. The MAPK signaling pathway is known to ultimately result in the dual phosphorylation and activation of terminal kinases, such as p38, c-Jun N-terminal kinases (JNKs), and extracellular signal-regulated kinases (ERK1/2 and ERK5), which are related to pressure-overload–induced cardiac hypertrophy [24]. Esposito *et al. *[24] mentioned the potential role of ERK activation in White Blood Cells (WBCs) as a novel molecular marker to identify uncontrolled human hypertension. In their study, JNK1 activation was also significantly induced in uncontrolled hypertension patients.

### Bipolar disorder data from the WTCCC GWA study

We also applied SNP-PRAGE to bipolar disorder (BD) data from the Wellcome Trust Case Control Consortium (WTCCC) which was established in 2005 to conduct GWA analysis for group of 50 research groups across the UK [25]. In our analysis, 1868 BD cases and 2938 controls were included and markers with high missing gene call rate (>5%), low MAF (<0.05) and significant deviation from Hardy-Weinberg equilibrium (P < 5.7 x 10^-7^) were excluded, leaving 354,093 SNPs. The logistic regression analysis with an additive model (1 *d.f.*) was conducted after adjustment for age, sex, region, and age x region.

SNP-PRAGE yielded 3 gene sets significantly associated with BD in terms of q-value at the 5% significance level (Table 3), while Z-statistic method did not detect any significant gene set (Table 4). The significant gene sets detected by SNP-PRAGE are AGPCR pathway, DREAM pathway, and CK1 pathway.

**Table 3:** WTCCC result: top 5 gene sets with smallest q-value associated with bipolar disorder phenotype from Z-statistic method

Gene set	No. genes	No. SNPs	p-value	q-value
EICOSANOID_SYNTHESIS	15	669	6.85 × 10^-4^	3.33 × 10^-1^
HSA04510_FOCAL ADHESION	171	10281	2.50 × 10^-3^	1.00
HSA01030_GLYCAN_STRUCTURES _BIOSYNTHESIS_1	91	7475	4.01 × 10^-3^	1.00
BADPATHWAY	17	1045	4.91 × 10^-3^	1.00
HSA05223_NON_SMALL_CELL_LUNG_CANCER	43	2933	5.49 × 10^-3^	1.00

**Table 4:** WTCCC result: top 5 gene sets with smallest q-value associated with bipolar disorder phenotype from SNP-PRAGE

Gene set	No. genes	No. SNPs	p-value	q-value
AGPCRPATHWAY	12	616	5.2 × 10^-5^	1.45× 10^-3^
DREAMPATHWAY	13	600	8.5× 10^-5^	1.45× 10^-3^
CK1PATHWAY	16	1079	3.1× 10^-4^	3.52× 10^-3^
BIOGENIC_AMINE_SYNTHESIS	16	914	1.0× 10^-3^	8.52× 10^-3^
BADPATHWAY	21	1045	5.6× 10^-3^	1.51× 10^-1^

AGPCR pathway is G-protein coupled receptors (GPCRs) signaling pathway which transduces extracelluar signals across the plasma membrane. In a genome-wide linkage survey, the region of chromosome 22q12 containing the GRK3 gene was identified as a susceptibility locus for BD in humans and GRK3 is expected to play an important role in the regulation of any one of many GPCRs [26]. DREAM is a multifunctional Ca^2+^-binding protein that can act as a transcriptional repressor for the prodynorphin gene. Subjects with BD were reported to show reduction of prodynorphin mRNA expression in discrete nuclei of the amygdaloid complex [27]. CK1 pathway is well known to be related to the circadian clock. Deregulation of this clock is involved in several human disorders. As a potent CK1ε inhibitor, a imidazole derivative, PF-670462 could be used for therapy of cognitive deficits in mood changes in bipolar disorders [28].

### Simulation study

In order to compare the performance of SNP-PRAGE with other GSA-GWA methods, we conducted the simulation study. Simulation data was generated based on a real GWA data. Using the subset of 5 gene sets from MsigDB canonical pathways, we constituted 5 gene sets so that each set has 20 genes. Over the gene sets, we varied the gene size which is the number of SNPs within a gene in order to study the effect of gene size on the gene set analysis. For example, one gene set consists of a small number of genes and other gene set consists of a large number of genes. The range of gene size is from 9 to 49 SNPs. Among 5 gene sets, we chose one causal gene set and selected 5 causal genes within the causal gene set. 500 individuals are randomly generated. For each causal gene, we selected one causal SNP whose minor allele frequency is about 0.2 for the selected individuals.

Given the genotype information of causal 5 SNPs and effect sizes, the case/control status was generated. Let *SNP_ij_* denote *j*th causal SNP in *i*th individual and *β* denotes effect size (=log odds ratio). Effect size of each causal SNP is given as 0, 0.3, or 0.6.

Simulated gene sets and their gene sizes are given in Table 5. Either set 1, set 3, or set 5 is used as the causal gene set. For each causal get set, 1000 simulation datasets were generated for the effect size 0 to compute type I error and 100 simulation datasets for effect size 0.3 and 0.6 to compute powers.

**Table 5:** Simulated gene set based on MsigDB pathways

Simulated gene set	No. genes	Gene size	Reference gene set
Set1	20	9~12 SNPs	HSA04060_CYTOKINE_CYTOKINE_RECEPTOR_INTERACTION
Set2	20	12~20 SNPs	HSA04010_MAPK_SIGNALING_PATHWAY
Set3	20	20~30 SNPs	HSA04810_REGULATION_OF_ACTIN_CYTOSKELETON
Set4	20	26~40 SNPs	HSA04510_FOCAL_ADHESION
Set5	20	36~49 SNPs	HSA04080_NEUROACTIVE_LIGAND_RECEPTOR_INTERACTION

**Figure 3:**
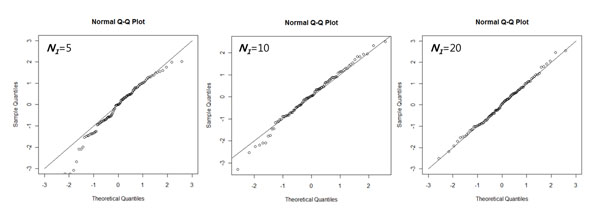
**QQ plot of set-level summary with various set size.** Under the assumption there is no causal set effect, Figure3 (a), (b), and (c) show the QQ plot of set summary with set size 5, 10, and 20, respectively.

In order to determine whether or not the central limit theorem works for relatively small gene set, we obtained a null distribution of set-level summary for reduced number of genes, say 5 and 10. We randomly chose 5 or 10 genes among 20 genes for each set. Figure 3 shows that the set level summary of small gene set follows a normal distribution when the number of genes is 10 and 20. However, there is a violation of normal approximation when the number of genes is 5. Thus, we expect that SNP-PRAGE would work well when the number of genes is at least 10. For practical applications, we recommend discarding the gene sets in the analysis if the number of genes is smaller than 10.

We compared the performance of SNP-PRAGE, Z-statistic method (Nam *et al*., 2010), modified GSEA method (Wang *et al*., 2007) and GLOSSI (Chai *et al*., 2009). We used the GenGen package for GSEA and the R package for other methods. SNP-PRAGE, Z- statistic method and GLOSSI use parametric test and GSEA method use nonparametric test with 1000 permutations. GLOSSI permute the data 100 times to consider the correlation of p-values resulting from LD among SNPs.

Type 1 error is defined as the proportion of cases whose p-values is less than the significance level when the effect size of causal SNP is zero. Power is defined as the proportion of cases whose p-value is less than the significant level when effect size of causal SNP is 0.3 and 0.6. Tables 6 and 7 summarize the type 1 error and power of the methods compared.

**Table 6:** Type 1 error (when effect size is 0) in simulation studies

Causal gene set	Gene set size	Gene size	Significance level	Z-statistic method					SNP-PRAGE					GLOSSI	GSEA
		
				*m*					*m*						
				1	2	3	4	5	1	2	3	4	5		
Set1	20 genes	9~12 SNPs	0.05 0.01	.005 .002	.003 .002	.004 .003	.004 .002	.003 .001	.057 .013	.053 .009	.054 .010	.054 .011	.053 .010	.052 .010	.051 .011

Set2	20 genes	20~30 SNPs	0.05 0.01	.083 .033	.087 .035	.084 .034	.080 .031	.080 .031	.051 .011	.052 .011	.052 .009	.050 .008	.052 .008	.051 .009	.049 .010

Set3	20 genes	36~49 SNPs	0.05 0.01	.430 .144	.641 .294	.760 .429	.864 .634	.891 .671	.047 .008	.049 .010	.050 .010	.050 .011	.051 .011	.049 .011	.052 .012

**Table 7:** Power (when effect size is 0.3 or 0.6) in the simulation studies

Effect size (=β)	Causal gene set	Gene set size	Gene size	significance level	Z-statistic method					SNP-PRAGE					GLOSSI	GSEA
		
					*m*					*m*						
					1	2	3	4	5	1	2	3	4	5		

0.3	Set1	20 genes	9~12 SNPs	0.05 0.01	.81 .78	.81 .75	.74 .66	.67 .55	.59 .38	.92 .90	.92 .91	.94 .92	.95 .92	.95 .91	.92 .89	.95 .91
	
	Set3	20 genes	20 ~30 SNPs	0.05 0.01	.85 .76	.78 .75	.79 .74	.79 .74	.76 .73	.81 .71	.81 .73	.83 .73	.82 .74	.83 .74	.82 .72	.83 .71
	
	Set5	20 genes	36~49 SNPs	0.05 0.01	.98 .95	.99 .97	.99 .97	.99 .99	.99 .98	.74 .61	.74 .62	.75 .62	.75 .62	.76 .63	.74 .60	.73 .61

0.6	Set1	20 genes	9~12 SNPs	0.05 0.01	.84 .80	.83 .75	.78 .69	.69 .60	.62 .48	.97 .94	.98 .95	.98 .97	.98 .97	.97 .96	.98 .96	.98 .97
	
	Set3	20 genes	20 ~30 SNPs	0.05 0.01	.86 .78	.89 .82	.86 .79	.88 .80	.88 .79	.84 .75	.85 .74	.86 .75	.86 .75	.87 .76	.84 .73	.85 .74
	
	Set5	20 genes	36~49 SNPs	0.05 0.01	1.0 .99	.99 .97	1.0 .99	1.0 .99	1.0 .99	.79 .69	.80 .71	.80 .72	.82 .72	.82 .73	.79 .69	.79 .68

Type 1 error and power of the Z-statistic depend largely on the gene size. When the causal gene set consisted of the genes with 9~12 SNPs, the Z-statistic method yielded low type 1 error and power. They tended to decrease, as *m* increased. We think it is because the genes with the smaller number of SNPs tend to have a larger minimum p-value and weaker LDs than the genes with a larger number of SNPs. When the causal gene set consists of the genes with 36~49 SNPs, on the other hand, the Z-statistic method yielded very high type 1 error and power. They tended to increase, as *m* increases. So the results from Z-statistic method can have high false positive errors, especially when the gene set has a larger number of genes.

On the other hand, SNP-PRAGE gave the consistent results irrespective of gene size. As *m* goes from 1 to 5, SNP-PRAGE gets a little larger power. Based on these results, it is desirable to use the mean of top *m* p-values instead of the minimum p-value as the gene-level measure. If the top *m* p-values are from the SNPs in LD, the method using the top *m* p-values can yield larger power than that using only the minimum p-value. The computed power based on SNP-PRAGE with appropriate *m* was similar but slightly larger compared to one of GLOSSI and GSEA. In SNP-PRAGE, type 1 error is near 0.05 at the significance level 0.05. Table 8 summarizes the computing time of each method. Z-statistic method has the fastest computing time, because LD structure between SNPs is not taken into account. SNP-PRAGE has the fastest computing time among the methods which consider LD between SNPs, Specifically, our simulation results show that GSEA and GLOSSI methods take 18.5 and 22.1 times, respectively, of computational efforts than SNP-PRAGE.

**Table 8:** Computing time for simulation data analysis

Process	Z-statistic method	SNP-PRAGE	GLOSSI (100 permutations)	GSEA (1000 permutations)
Single SNP analysis	40sec	40sec	34 min	26 min 15sec
Gene set analysis	0.3 sec	52sec	0.5sec	2 min 10sec
Total analysis	40.3sec	1min 32sec	34 min 0.5sec	28 min 25sec

The single SNP analysis for the Korean GWA data requires more than 1000 computing time compared to one set of simulation data. So, it would take a very long period of time if GSEA and GLOSSI are applied to our data, because both methods require permutation process. Thus, in practice it would not be easy to handle a large scale GWA data by GSEA and GLOSSI.

## Discussion

The power of SNP-PRAGE was computed for the several choice of *m*. When we choose appropriate *m* for the gene-level summary, the computed power based on SNP-PRAGE was similar but slightly larger compared to one of GLOSSI and GSEA in the simulation study. Then how can we choose the appropriate *m* for the gene-level summary?

The best choice for the number of the top p-values used in gene-level summary depends on the LD structure among the SNPs within the causal genes. While we set a fixed *m* over the genes for the summary in SNP-PRAGE, setting different *m* over the genes according to each effective gene size can be considered in the future study.

Our SNP-PRAGE can be extended in several ways. In this study, we assume the gene sets are independent from each other. However, the gene sets often share some common genes because one gene can have multiple biological functions. So handling the overlapped common genes between gene set is another challenging issue.

SNP-PRAGE method is based on a normal distribution and similar to ANOVA (Analysis of Variance) model. In fact, SNP-PRAGE can be expressed as ANOVA model with some contrast and modified estimation of variance. As an extension, another well-defined parametric model can be applied. A nested ANOVA can be applied to the gene set analysis in terms of that gene effect is nested within gene-set effect. A mixed effect model can also be applied by treating the gene specific effects as random effects. Addressing these challenges we expect a more powerful GSA-SNP method in our near future.

## Conclusions

Single SNP analysis in GWAS offers only a limited understanding of complex diseases because the complex disease often arises from the joint action of multiple genetic variants. Single SNP analysis can find only a few most significant SNPs. GSA-GWA increases the power to detect the genetic variants which have a weak association but a meaningful biological association with a phenotype .GSA-GWA methods test the significance of gene set via permutation by generating permuted data more than hundred times, which requires expensive computational efforts. The use of a parametric test can reduce the computing time, because it needs to calculate the gene set statistic only once.

We compared the performance and computing time of three parametric test-based GSA-GWAs (Z-statistic method, GLOSSI, SNP-PRAGE) and one nonparametric test-based GSA-GWA (GSEA) in simulation study. The Z-statistic method does not consider the LD and has the shortest computing time but may have lots of false positive results because of overestimated gene set statistics when the gene set has many large genes. GLOSSI uses a parametric test but it needs to permute phenotype 100 times for an estimation of the correlation between association measures and GSEA requires much more permutations than GLOSSI. SNP-PRAGE reduces computing time much and has comparable performance to GLOSSI and GSEA without going through the permutation step.

We found that consideration of LD blocks between SNPs helps us to deal with the correlation between p-values more appropriately. The approach based on the mean of top *m* p-values provides more consistent and stable result than the approach based on the top *m*th p-value. Multiplying the effective gene size to the minimum p-value for the gene-level summary of SNP-PRAGE can reduce the false positive errors when the gene size is large. We expect the SNP-PRAGE to play an important role in the parametric gene set analysis of large-scale GWA data.

## Competing interests

The authors declare that they have no competing interests.

## Authors’ contributions

JL designed the summarization algorithm and drafted the manuscript. SA provided general trends of gene set analysis and drafted some of background part. SO and BW critically read the draft and contributed to the design of the algorithm. TP coordinated the work and help to draft the manuscript.
